# Novel Strategies for Cancer Treatment: Highlights from the 55th IACR Annual Conference

**DOI:** 10.3390/cancers11081125

**Published:** 2019-08-07

**Authors:** Sara Charmsaz, Denis M. Collins, Antoinette S. Perry, Maria Prencipe

**Affiliations:** 1 Endocrine Oncology Research Group, Department of Surgery, Royal College of Surgeons in Ireland, D2 Dublin, Ireland; 2 National Institute for Cellular Biotechnology, Dublin City University, Glasnevin, D9 Dublin, Ireland; 3 Cancer Biology and Therapeutics Laboratory, UCD Conway Institute, University College Dublin, Belfield, D4 Dublin, Ireland; 4 UCD School of Biology and Environmental Science, University College Dublin, Belfield, D4 Dublin, Ireland; 5 UCD School of Biomolecular and Biomedical Science, University College Dublin, Belfield, D4 Dublin, Ireland

**Keywords:** cancer therapeutics, combination therapy, immune therapy, epigenetics, physical exercise, liquid biopsies, multi-omics

## Abstract

While conventional cancer treatments, such as surgery, radiotherapy and chemotherapy, have been combined for decades in an effort to treat cancer patients, the emergence of novel fields of cancer research have led to a renewed interest in combining conventional treatments with more innovative approaches. The realisation that cancer progression is not exclusively due to changes in the cancer epithelial cells, but also involves changes in the tumour microenvironment, has opened new avenues for combination treatments. Here we discuss the use of combination therapies presented at the 55th Irish Association for Cancer Research (IACR) Annual Conference, highlighting examples of novel therapeutic strategies which, combined with conventional therapies, may greatly enhance not only the overall outcome for patients, but also the quality of life for cancer survivors. Among the novel treatment strategies, immune metabolism, epigenetic therapies and physical exercise are presented. In addition, novel technologies in the field of precision medicine, which will be useful to discover new therapeutics and to stratify patients for combination treatments, are also discussed.

## 1. Introduction

For many decades, cancer treatment was limited to only a few options for patients. These included surgery and radiation therapy for solid localized tumours, and chemotherapy for blood-related cancers and solid metastatic tumours. These therapies have been used as single treatments or in combination for a long time. Recently, with the advent of targeted therapies, a big emphasis has been put on the biological mechanisms underlying response/resistance to targeted agents. As a result, our understanding of the many pathways involved in cancer progression and the ways in which they can be targeted has improved dramatically, with combinatorial strategies involving multiple targeted therapies or “traditional” chemotherapeutics, such as the taxanes and platinum compounds, being found to have a synergistic effect [1]. However, while conventional therapies, such as targeted therapies, radiation therapy and chemotherapy, mainly target epithelial cancer cells, we now know that cancer progression is not exclusively due to changes in cancer cells, but also involves the tumour microenvironment (TME), as well as alterations in cellular metabolism and immune response, offering new avenues for cancer therapies. The use of immune therapy in the treatment of cancer has gained traction over the last few years, culminating in the recent Nobel Prize for Physiology or Medicine to Prof. James Allison and Prof. Tasuku Honjo for their seminal work in this field [2]. Their work has established negative immunomodulation through the inhibition of immune checkpoint proteins, such as Cytotoxic T-lymphocyte-associated Protein 4 (CTLA-4) and Programmed Cell Death Protein 1 (PD-1), as a cornerstone of modern cancer treatment. Immune checkpoint inhibitors, including ipilimumab (anti-CTLA-4) and pembrolizumab (anti-PD-1), are in trial in multiple cancer types, moving from single agent studies to combinatorial studies with other immune checkpoint inhibitors and more classical chemotherapies [3,4]. Epigenetics drugs such as 5-Azacytosine have now established their presence in the clinic for blood-related malignancies [5] and can be used in combination with traditional treatments in solid tumours where they re-sensitize cancer cells to certain types of chemotherapy [6,7]. Interestingly, hypomethylation of the promoter regions of CTLA-4 and PD-1 have been associated with increased expression of these genes in the TME in lung cancer [8]. Although exercise is not a pharmacologic intervention, it does confer drug-like effects that cause changes to the individual’s homeostasis. The importance of exercise in the cancer journey has been recently highlighted in a report by the Clinical Oncology Society of Australia, with the clear recommendation that exercise should be embedded as part of standard practice in cancer care [9]. Multi-omics technologies (genomic, epigenomic, transcriptomic, epi-transcriptomic and proteomic networks) offer powerful new tools to identify novel therapeutic targets and associated companion diagnostics [10]. Here, we report a snapshot of the more innovative combination therapies presented at the 55th Annual Conference of the Irish Association for Cancer Research (IACR).

## 2. Cancer and Immune Metabolism

The use of immunotherapy in the treatment of cancer has received considerable attention in recent years. Natural killer cells (NKs) are members of the innate lymphoid cell population and, as their name suggests, they have a role in eliminating cells that are known to be dangerous to the host organism, including cancer cells, viral-infected cells and foreign cells [11]. Prof. David Finlay’s group from Trinity College Dublin (TCD) has focused on understanding how cellular metabolism and the fuels available in the microenvironment control NK cell metabolism and facilitate their effector function.

Studies by Prof. Finlay’s group have shown that the cellular fuels available to immune cells have a big impact on their function. They found that in cytokine-activated NK cells, robust induction of glycolysis and oxidative phosphorylation (OXPHOS) are essential for effective NK cell anti-cancer functions [12]. Their group identified the key metabolic regulators of this response to be mammalian target of rapamycin complex 1 (mTORC1), cMyc and sterol regulatory element-binding protein (SREBP) [13]. In cancer and other diseases, impaired cellular metabolism can lead to dysfunctional NK cells. In cancer, low levels of glucose may result in direct or indirect inhibition of NK cell metabolism through alteration in the activity of nutrient-sensing signalling pathways [13]. In a metabolically restrictive tumour microenvironment where tumour cells consume large quantities of fuels, the anti-tumour immune response is suppressed [13]. New strategies have been introduced to modulate NK cell function in the tumour microenvironment through modulation of its metabolic requirements. One strategy is the use of chemotherapy/radiotherapy alongside immunotherapies to reduce the number of fuel-consuming tumour cells, by inducing tumour cell death and increasing glucose levels required for the anti-tumour response of the NK cells. On the other hand, inhibition of glutaminase will reduce glutamine consumption and increase the glutamine available for the metabolic activity of NK cells [13]. Other strategies involve the use of metabolic agents in combination with checkpoint inhibitor antibodies. These include the use of anti-PD-1, anti-CTLA-4, or anti-PD-L1, resulting in reduced T-cell glycolysis and increased glucose levels in the TME and, in particular, an increase in NK cells’ anti-tumour effect [14]. Depletion of other nutrients can also have an effect on the glycolytic rate of the immune cells. Expression of the enzymes indoleamine-pyrrole 2,3-dioxygenase (IDO) and arginase-1 by tumour cells results in the depletion of tryptophan and arginine, which can inhibit T-cell and NK cell function, and therefore inhibition of these enzymes with metabolic agents can result in an increased antitumour immune response [14]. In summary, the studies conducted by Prof. Finlay’s group suggest that cellular metabolism is essential for the normal function of NK cells, and could potentially be considered as a new therapeutic strategy in combination with immunotherapy for the treatment of cancer. Considering that immunotherapies still fail in many patients because of insufficient reprogramming of the immunosuppressive TME, Prof. Finlay’s studies will contribute to elucidate some of the mechanisms by which drugs targeting cancer metabolism might synergistically enhance immunotherapy via metabolic reprogramming of the TME.

## 3. Epigenetic Therapies

The 5-azacytosine DNA methyltransferase inhibitors (DNMTis) have established a presence in the clinic for the treatment of myelodysplastic syndrome and acute myelogenous leukaemia. These agents act as S-phase specific inhibitors of the DNA methyltransferase enzymes and cause global decreases in DNA methylation [15]. Haematological patients respond to DNMTis as monotherapies, but the reasons for doing so are not entirely clear. It will be important to determine the exact mechanism(s) of action of these epigenetic agents in order to increase their efficacy and broaden their scope within solid tumours. Additionally, the use of DNMTis in solid tumours requires the use of combination therapies to increase patient responses. Professor Peter Jones, Chief Scientific Officer at Van Andel Research Institute Grand Rapids, Michigan, U.S., and a pioneer in the field of epigenetics, discussed some of the potential mechanisms by which DNMTis function to cause responses. Traditionally, the main explanation for the effects of epigenetic therapies was that they upregulate the expression of abnormally silenced tumour suppressor genes, thus resulting in the restoration of growth control to treated cells [16,17]. Most recently, Professor Jones’ team has become interested in the roles of sequences constitutively methylated in both normal and cancer cells as targets for DNMTis. For example, the removal of DNA methylation from gene bodies can result in decreased transcription, leading to lower levels of transcription factors such as MYC proto-oncogene, which are commonly upregulated in cancer. This results in substantial downregulation of proto-oncogenes in the MYC pathway [18]. DNMTis are also powerful inducers of human endogenous retroviruses (ERVs). ERVs are a class of transposable elements that are acquired when retroviruses infect germ cells during evolution. The main mechanism for silencing these ERVs is DNA methylation. Therefore, activation of the ERVs through demethylating agents can lead to a state of viral mimicry in which the treated cancer interprets the induced ERV expression as being due to an infection by an exogenous virus and mounts an innate immune response, leading to production of type I and type III interferon and other cytokines [19,20]. This results in decreased cancer cell fitness and attraction of cytotoxic T lymphocytes (CTLs) to the TME. These infiltrating immune cells also show epigenetic abnormalities and can therefore be targeted by epigenetic drugs. For example, the CTLs become exhausted when continuously stimulated by the TME. The exhausted phenotype is characterised by aberrant DNA methylation of genes involved in T-cell effector function; therefore, DNMTis may be used to reprogramme the CTLs into an effector phenotype [21]. This important mechanism of action of epigenetic drugs highlights the potential for combining these agents with the use of checkpoint inhibitors in solid tumours to capitalize on the viral-defense pathways induced. At this regard, several studies, reviewed by Zahnow et al. [22], offered a rationale for combining epigenetic drugs, such as 5-Azacytidine (DNMTi) and entinostat (HDACi), with checkpoint inhibitors, such as anti-CTLA-4 and anti-PD1 agents. Efficacy of this combinatorial regimen has been demonstrated in several cancer types, including colorectal, breast, prostate, renal, ovarian cancers and melanoma [22]. The combination of various agents which might increase the efficacy of DNMTi treatment directly such as the inclusion of Vitamin C in the treatment regimen was also discussed. While Vitamin C deficiency is rare in the general population, cancer patients show low levels of it [23], suggesting that Vitamin C supplements could be beneficial for cancer patients. The role of Vitamin C in enhancing the viral mimicry resulting from epigenetic treatments [24] is due to the fact that Vitamin C is an essential cofactor for the ten-eleven translocation (TET) enzymes, which are actively involved in DNA demethylation [25]. Experimental evaluation of the combination of Vitamin C with 5-aza-2’-deoxycytidine in preclinical models [24] suggested that a strong synergy could be expected in patients [26], and clinical trials designed to test this are currently underway.

## 4. When Exercise Is the Drug: Can Activity Levels Really Be Used to Treat Cancer?

The importance of exercise in the cancer journey was recently highlighted in a report by the Clinical Oncology Society of Australia, with the clear recommendation that exercise should be embedded as part of standard practice in cancer care [9]. Pre-operative exercise optimises physical fitness, enabling an individual to maintain better overall health during and after surgery. It has been shown to significantly improve fitness and health-related quality of life (HRQoL) [27,28,29,30]; however, much of this work has been reported following hospital-based [27,28] and some home-based [29,30] programmes. Community-based exercise programmes are attractive, as they represent a more accessible, scalable and sustainable alternative to hospital-based programmes, and may reduce the burden on the healthcare system. Although few studies have explored community-based training in the pre-operative setting, the early data are encouraging, showing feasibility and effectiveness [31,32].

Dr. Noel McCaffrey, medical director of ExWell (a community-based chronic rehabilitation exercise service) and his colleague Dr. Lisa Loughney (PhD.), along with local consultant surgeons (Mater and Beaumont hospitals in Dublin, Ireland), are conducting a programme of work investigating community-based (in a gym facility) exercise programmes in the pre-operative setting. Their first pilot study investigated the compliance, adherence and effectiveness of a community-based pre-operative exercise programme in people with a newly diagnosed prostate cancer and colorectal cancer (CRC) scheduled for surgery. Thirty-two surgical oncological (15 prostate cancer and 17 CRC participants) were recruited and assessed to measure health-related (HR) components of fitness (strength and functional exercise capacity) and HRQoL. An exercise programme was prescribed in the time available prior to surgery with repeat assessments pre-operatively. Exercise training was delivered over a median interquartile range (IQR) of 4 (3–4) weeks and 2 (1–3) weeks for the prostate cancer and CRC participants with >80% adherence. This pilot study showed that participants had acceptable compliance and adherence rates to the community-based pre-operative exercise programme and that it significantly increased lower body strength and HRQoL.

A qualitative sub-study, as part of the above pilot study, investigated the effects of the pre-operative exercise programme on perceived wellbeing and HRQoL in the prostate cancer group. Following completion of the exercise programme (within 1 week before surgery), 11 participants took part in a semi-structured interview which covered four broad HRQoL domains, including physical, psychological, social and spiritual wellbeing. Findings showed that engagement in the pre-operative exercise programme provided participants with: (1) a teachable moment; (2) acted as a vehicle to recovery; (3) a sense of optimism and (4) social connectedness. This qualitative study showed that the exercise programme enhanced wellbeing and improved perceived HRQoL. Further research is required to explore this in a larger, adequately-powered sample.

The research group have just completed a phase 1 study examining the feasibility and effectiveness of a community-based pre-operative exercise programme (initiated immediately after cancer diagnosis) in people with oesophageal and gastric cancer and scheduled for neo-adjuvant cancer treatment (NCT) followed by surgical resection. The rationale for this research study is based on previous research that showed NCT significantly reduces physical fitness prior to surgery [33,34]. Low pre-operative fitness levels may compromise a patient’s ability to undergo surgical resection and are associated with poor post-operative outcomes [33,34]. Eight patients (six male and two female) were recruited to participate in this phase 1 study. An exercise programme was prescribed before, during and after NCT, and continued until surgical resection with repeat assessments post-NCT and pre-surgery. Findings showed that this community-based exercise programme is feasible and improves HR components of fitness and HRQoL. These data informed the design of the PERIOP-OG trial (recruitment start date: March 2019), a pragmatic multi-centre, randomised controlled trial, investigating the benefits of a community-based exercise training programme compared to standard usual care (no formal exercise) in the same patient group (clinicaltrials.gov identifier: NCT03807518).

In keeping with the theme of the importance of physical exercise in the cancer journey, Dr. Gillian Prue and her team from Queen’s University Belfast (QUB) adopt a precision oncology approach utilising exercise as an anti-cancer treatment. There is sufficient evidence demonstrating the favourable effects of exercise on symptom control and quality of life (QOL) in prostate cancer, such as in countering the effects of androgen deprivation therapy [35,36,37]. Epidemiological studies have suggested that exercise may improve disease-specific and overall survival in prostate cancer [38,39]; however, this has yet to be demonstrated in a clinical population.

Many exercise oncology trials adopt a generic, linear approach to training (i.e., low to medium intensity gradually increasing over time), but to maximise outcome, the ‘principles of training’ —traditionally used for athletes—should be applied to exercise prescriptions [40]. The use of generic exercise prescription, though successful in some cases, has led to homogenous exercise programmes being prescribed for largely heterogeneous populations, not taking into consideration the unique needs and preferences of the individual. This may mask the full therapeutic potential of the exercise programme, prompting calls for the potentially more effective non-linear models which focus on individualisation, specificity, progressive overload and recovery. This approach involves manipulating intensity, duration and occasionally the frequency of training sessions to allow the training volume to continually increase across the entire programme. As there is considerable heterogeneity in cancer progression and treatment, exercise programming should be equally individualised, to promote safety and optimise the efficacy of treatment for the individual.

Dr. Gillian Prue and her team examine the survival advantage that can be achieved through targeted, tailored, exercise medicine. This is currently being achieved via a global research trial being led by Prof. Rob Newton and colleagues in Edith Cowan University, Perth, Australia. This Movember-funded study, which is part pf the Movember Global Action Plan (GAP4), is entitled INTERVAL–MCRPC (intense exercise for survival with metastatic castrate-resistant prostate cancer), a multicentre, randomised, controlled, phase III study [41]. The trial is designed to test the effects of exercise on prostate cancer progression and treatment side effects.

In addition, to provide evidence on the feasibility of exercise interventions in patients for whom high intensity exercise is not suitable, Dr. Prue’s team are currently running a parallel Northern Ireland-specific study based upon an exercise intervention (Exercise for Advanced Prostate Cancer: a Multicomponent Feasibility Trial (EXACT) and CRC trial) developed and recently tested in CRC survivors by colleagues from Ulster University. EXACT-MCRPC (EXACT-metastatic castrate-resistant prostate cancer) offers the benefits of participating in a multicomponent physical activity programme to those men with MCRPC who are ineligible for the INTERVAL programme that contains high intensity exercise. The aim is to ensure that all men have the opportunity to capitalise on the benefits of increased physical activity by offering a lower intensity lifestyle physical activity intervention. This is the first of its kind in this advanced and unwell population. This feasibility study is providing preliminary evidence on the acceptability, feasibility and efficacy of moderate intensity physical activity among men with very advanced cancer, and setting the benchmark for all other cancer patients.

Dr. Prue’s team is also planning to extend this research programme to pancreatic cancer. Unlike other gastrointestinal cancers (such as CRC), epidemiological evidence of the association between level of physical activity and pancreatic cancer risk and/or progression remains limited, but some evidence suggests greater volumes decrease risk [42,43]. Given the many other benefits of exercise, if there is the potential that exercise can impact survival in this group, given their poor prognosis, it should be investigated. There are a small number of RCTs, with the vast majority utilising the generic, linear approach of home-based exercise, and only one focusing on patients’ post-resection [44,45,46,47]. There is some evidence in the form of case studies that demonstrate patients with pancreatic cancer can undertake the necessary type of exercise required to maximise the therapeutic effect, i.e., through structured, high intensity, supervised combined (aerobic and resistance) exercise during chemotherapy without any adverse effects. Dr. Cormie and colleagues reported a combined exercise programme, following surgery and during adjuvant therapy, which improved not only physical capacity, QOL, fatigue, sleep quality and distress, but also prevented muscular atrophy [48]. Given that body composition has been cited as a predictor of toxicity [49] and pancreatic cancer patients commonly suffer rapid post-surgery weight loss and cachexia, this result is of clinical relevance. A second case study demonstrated that combined high intensity exercise, following surgery and while receiving adjuvant therapy, was well-tolerated and feasible, and at the same time resulted in maintaining body weight and improving strength and aerobic capacity [50]. Exercise-induced physiological improvements appear to aid treatment tolerance, mitigate toxicities and arguably facilitate in maximising treatment doses. On the basis of this evidence, Dr. Prue’s team are planning to conduct a single group feasibility study that will be used to test the acceptability of the prescribed exercise programme, recruitment and retention rates, the acceptability of outcome measurement and provide useful data for the calculation of an effect size for a larger trial.

## 5. Multi-Omics as a Novel Tool for Discovering New Therapeutics for Cancer

Human biological processes are driven by a complex network of events leading to specific functional phenotypes. These include genomic, epigenomic, transcriptomic, epi-transcriptomic and proteomic networks that cooperate together to deliver a specific biological function. New technologies, as well as advances in analytical techniques, have revolutionized ‘omic’ science and allowed for a more in-depth and integrative understanding of biological processes that lead to various diseases including cancer [51]. The European Association for Cancer Research (EACR) Senior Investigator Award Winner, Dr. Sara Charmsaz from the Endocrine Oncology Research group (EORG) in Royal College of Surgeons Ireland, described their efforts in integrating ‘omic’ data to identify new therapeutic targets for treatment of endocrine-resistant breast cancer. Dr. Charmsaz presented studies that integrated proteomic and transcriptomic data to identify new therapeutic targets, as well as novel companion diagnostics tools, for Estrogen Receptor (ER)-positive breast cancer. Two main targets were identified, A Disintegrin And Metalloprotease domain 22 (ADAM22) as a potential new therapeutic and S100 calcium-binding protein β S100β as a companion diagnostic for early identification of patients at risk of developing metastasis. S100β as a companion diagnostic is used to identify patients at risk of developing metastatic disease and suggests a src-kinase inhibitor (Dasatinib) as a potential new therapeutic used in combination with endocrine therapy in patients with elevated levels of S100β [52,53]. More recently, they have used transcriptomic data, not only from primary and matched metastatic patients but also from patients with good prognosis and patients with poor outcome, to find new targeted therapies [54,55,56]. These efforts included a new study where RNA-sequencing and DNA methylation data were integrated to test the potential of novel therapeutics including a DNMTi (RG108) for the treatment of aggressive metastatic breast cancer [54].

Dr. Charmsaz is currently focused on understanding epi-transcriptomic alterations in ER-positive endocrine-resistant breast cancer and is in the process of integrating these data with proteomics to identify novel therapeutic targets for treatment of aggressive breast cancer.

## 6. Discussion and Concluding Remarks

In the past decades, we witnessed tremendous advances in cancer research which have opened new and exciting avenues for the future of cancer treatment and management. Combination therapies certainly played a huge part in this process. Each plenary session at the 2019 IACR meeting covered innovative approaches to cancer therapy which combine traditional therapies with novel ways of targeting cancer progression, summarised in Table 1. Some of these approaches, such as epigenetic treatments, have been studied and developed for a long time and are already incorporated into clinical practice for blood-related malignancies as well as being actively investigated in combination with conventional therapies in solid tumours [16]. In addition, further understanding of the mechanisms underlying epigenetic drugs reveals a possible link with enhancing patients’ immune response [57]. This new mechanism of action highlights the powerful effect of drug combinations in simultaneously targeting several biological processes relevant to cancer progression. The combination of immune therapy with conventional therapy is becoming mainstay for many cancers [58] and has revolutionized the outcome of traditionally difficult-to-treat cancers such as lung cancer [59]. Moreover, a number of clinical trials are ongoing to test metabolic drugs in combination with immune-checkpoint inhibitors in several cancer types [60]. While physical exercise is not currently being used in specific combination therapies, it is emerging as a very promising intervention for cancer management [9] and will undoubtedly gain centre stage in the future as a novel approach to target cancer cells. While still in its infancy as a “targeted” cancer treatment, recent clinical trials are already testing the efficacy of exercise as a targeted medicine [61] with a view of incorporating it as an additional treatment to conventional therapies. Combining ‘omics’ data from different sources is becoming a powerful tool for disease-relevant target discovery.

The combination therapies presented at the 2019 IACR Conference highlighted the latest findings in the field of cancer therapeutics. These innovative approaches will offer additional therapeutic options to cancer patients. While more research is needed to refine our understanding of the molecular mechanisms underlying drug combinations, the development of ‘omics’ technologies will allow us to better predict the response to combination therapies, thus improving our ability to use them in the clinic.

## Figures and Tables

**Table 1:** Contributing speakers and highlights of their talks.

Speaker	Affiliation	Title of Talk	Highlights
Prof. David Finlay	Trinity College, Dublin, Ireland	Fuelling robust anti-tumour natural killer (NK) cell responses	Metabolic drugs might synergistically enhance immunotherapy via metabolic reprogramming of the tumour microenvironment (TME).
Prof. Peter Jones	Van Andel Institute, Grand Rapids, Michigan, U.S.	Epigenetic therapies	Epigenetic treatments are already incorporated into clinical practice for blood-related malignancies and are actively investigated in combination with conventional therapies in solid tumours. Further understanding of their mechanisms also reveals a possible link with enhancing patients’ immune response.
Dr. Noel McCaffrey	Dublin City University, Ireland	Community-based exercise in cancer survivorship	While physical exercise is not currently being used in specific combination therapies, it is emerging as a very promising intervention for cancer management and will undoubtedly gain centre stage in the future as a novel approach to target cancer cells.
Dr. Gillian Prue	Queen’s University, Belfast, Northern Ireland	When exercise is the drug: can activity levels really be used to treat prostate cancer?	
Dr. Sara Charmsaz	Royal College of Surgeons in Ireland, Ireland	RNA-methylation in Estrogen Receptor (ER)-positive breast cancer	The development of ‘omics’ technologies will allow us to better predict the response to combination therapies, thus improving our ability to use them in the clinic.
